# Supplementary material for: Integrating mass spectrometry with MD simulations reveals the role of lipids in Na+/H+ antiporters
Source: Nat Commun. 2017 Jan 10;8:13993. doi: 10.1038/ncomms13993 (PMC5234078; doi:10.1038/ncomms13993)
Supplement: Supplementary Information — Supplementary Figures and Supplementary Tables [file ncomms13993-s1.pdf]

Table S1. Masses of the human, archaeal, and bacterial antiporters used in this study. Masses were determined from the centroids of the three most abundant charge states. For the NhaA dimer, no suitable charge state series could be detected.

Protein	Calculated mass	Measured monomer	Measured dimer
NapA	41149.3	41174.5 ± 1.9	82350.4 ± 3.8
NHA2	43483.1	43510.7 ± 14.4	87026.6 ± 24.4
NhaA	42900.4	42899.6 ± 0.2	-

Table S2. Theoretical CCS values for NapA. Values for the crystal structure (1) and the energy-minimized gas phase structure (this study) were calculated using the projection approximation (PA) method as implemented in IMPACT, the PA method including an experimentally derived scaling factor (12), and the PA method including a scaling factor for the trajectory method (TJM) (10). All values are given in $Å^2$.

Structure	PA	PA × Scaling factor	PA × TJM factor
Crystal structure (4BWZ)	4299	4998	5564
Gas phase MD model 300K	3889	4522	5008

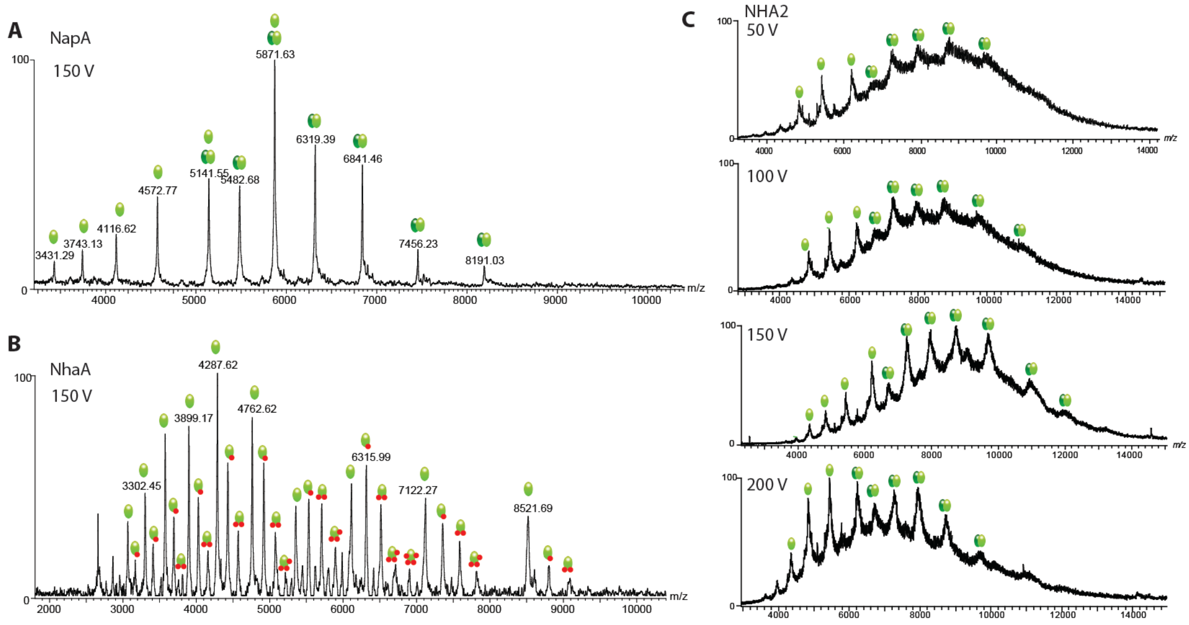

Figure S1. MS spectra of NapA (A) and NhaA (B) released from C12E9 at a collision voltage of 150 V. While NapA remains mostly dimeric, NhaA dimers are dissociated completely. (C) Like NapA, dimers of the human NHA2 remain mostly intact across the entire activation energy range. Notably, increasing the collision energy strips adducts off the dimers, demonstrating that the high baseline observed at low activation energy is caused by adduct binding to dimeric NHA2.

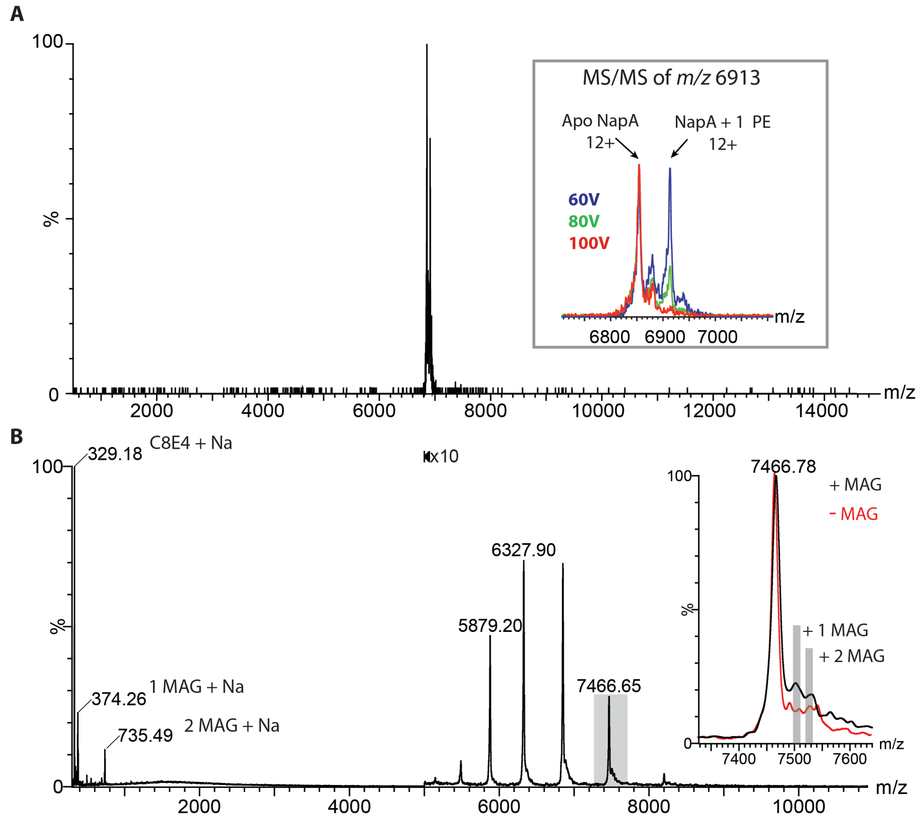

Figure S2. (A) MS/MS of NapA-PE complexes shows lipid dissociation in response to collisional activation. **(B)** 9.9 MAG readily dissociates with the detergent, leaving only minor amounts bound to NapA (insert) at a collision voltage of 20 V.

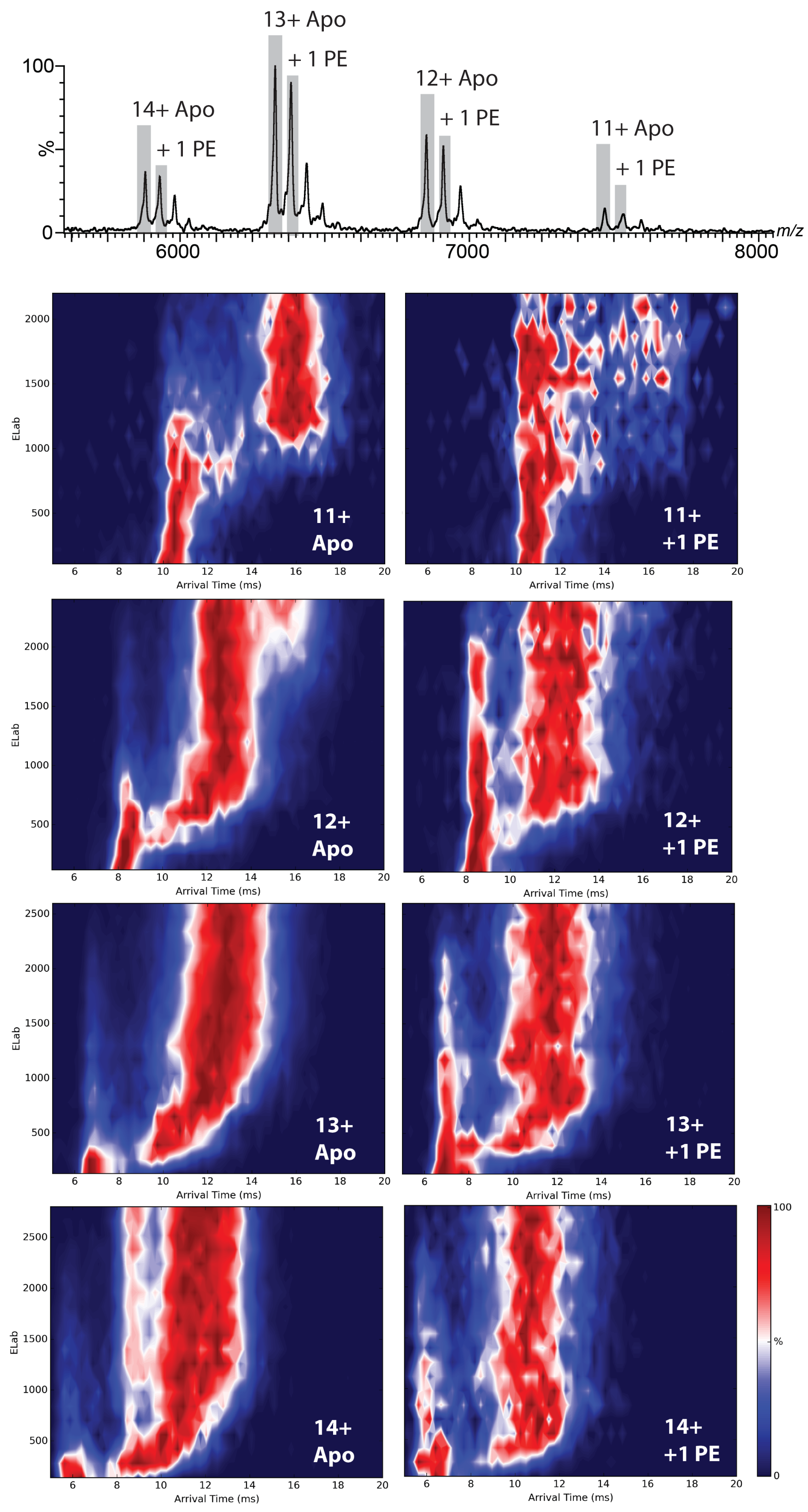

Figure S3. Lipid-mediated stabilization and lower arrival times of the unfolded state is observed for all charge states.

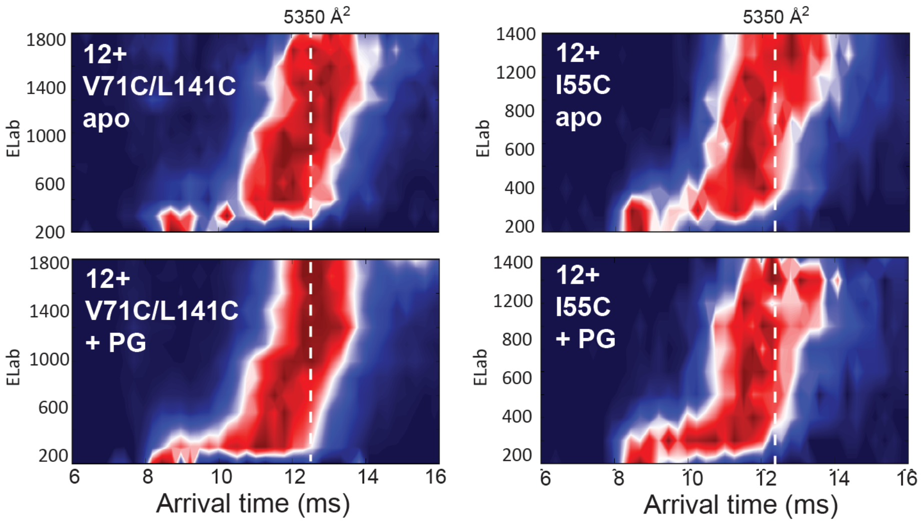

Figure S4. Unfolding trajectories of the 12+ charge state of disulphide-linked V71C/L141C and I55C NapA show that lipid binding does not reduce the CCS of the unfolded states of the disulphide-linked variants.

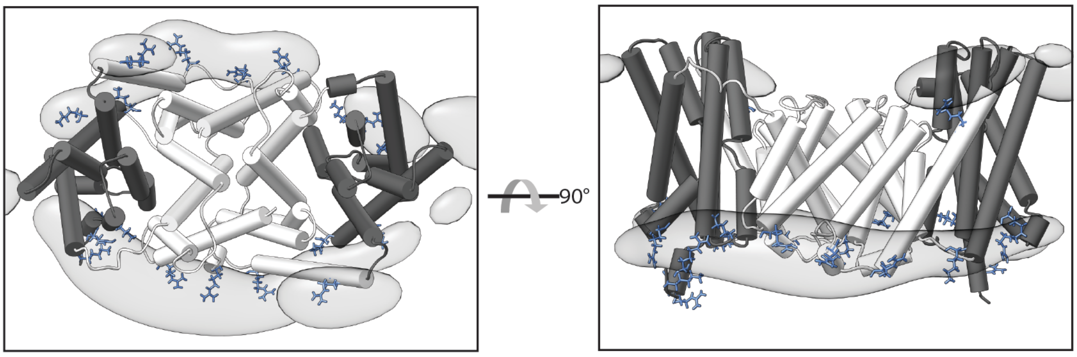

Figure S5. Lipid head-groups cluster around basic residues in NapA. In MD simulations of NapA in a PE bilayer, the highest local phosphate head-group densities (grey areas) are observed around positively charged residues (blue) located on the dimer domain. Dimer and core domains are shown in white and black, respectively.

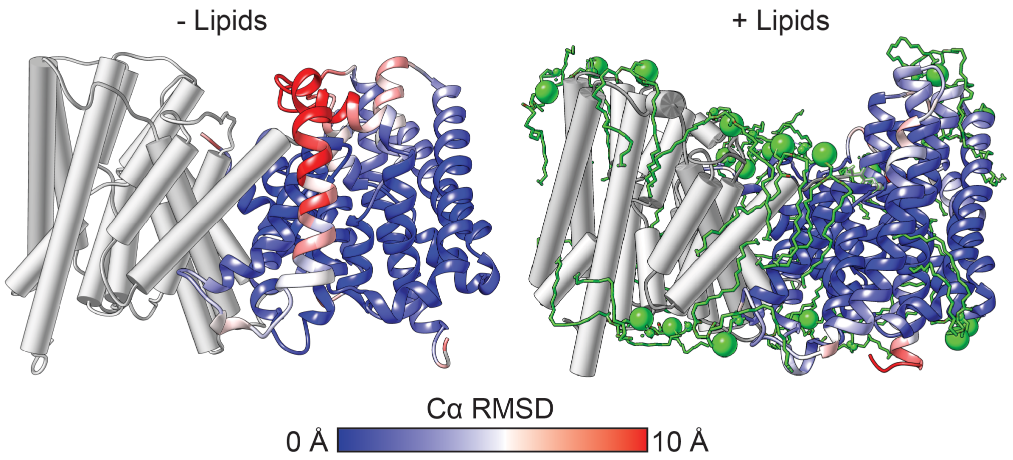

Figure S6. Distribution of Cα RMSDs of lipid-free and lipid-bound NapA after 20 ns equilibration in vacuum show conformational changes relative to the structure equilibrated in a PE bilayer.

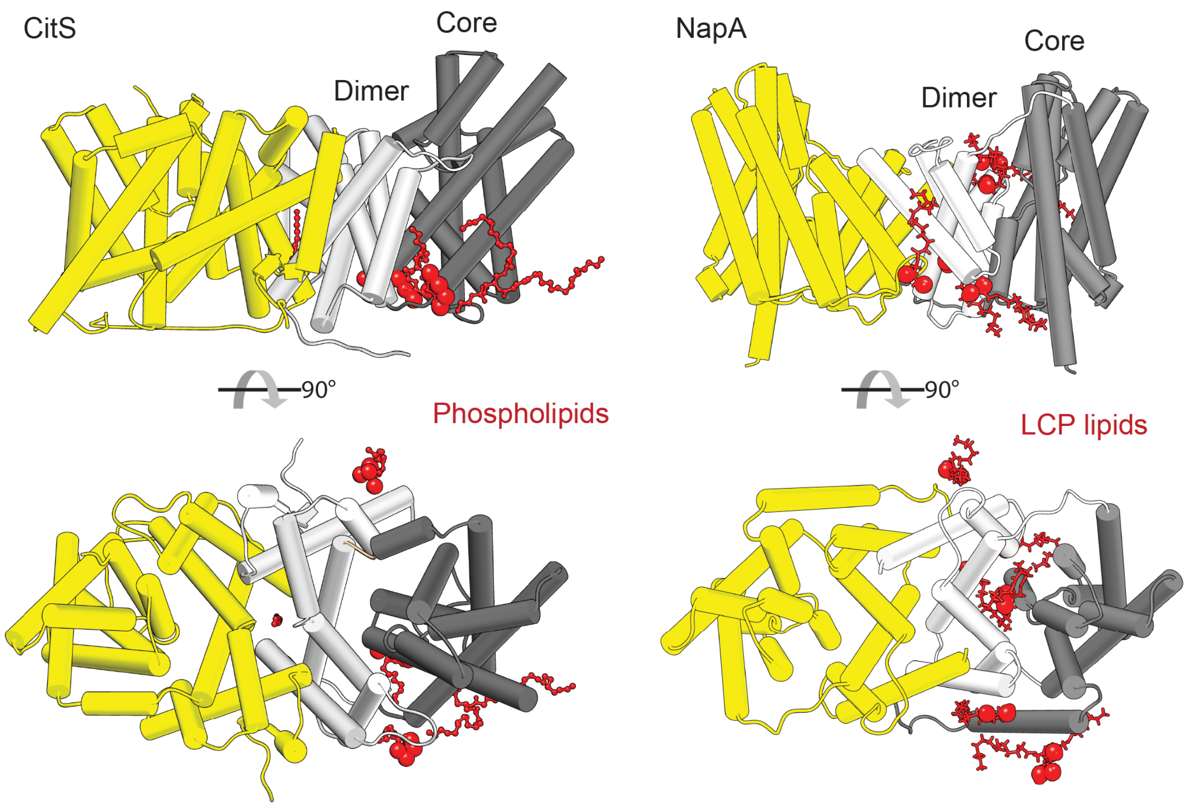

Figure S7. Comparison of lipid locations in CitS and NapA. In the citrate symporter CitS, co-crystallized phospholipids are located close to the core-dimer interface. Similarly, the lipid cubic phase structure of NapA shows the LCP lipid 7.7 MAG inserting into the space between core and dimer interface. In both cases, the lipid headgroups (spheres) are oriented towards the connecting segments between the domains. One dimer subunit is rendered in white (dimer) and black (core), the second dimer subunit is rendered in yellow.

A

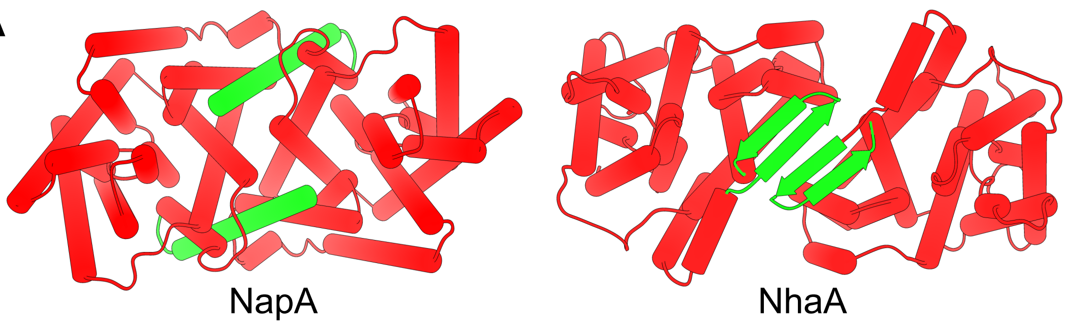

B

```
NhaA ————————————————————————————————————————————————————————————
NapA ------------------------------------------------------------
NHA2 MGDEDKRITYEDSEPSTGMNYTPSMHQEAQEETVMKLKGIDANEPTEGSILLKSSEKKLQ

                                                       TM -1
NhaA ————————————————————————————————————————————————————————————
NapA ----------------------------------------MHGAEH-----LLE---
NHA2 ETPTEANHVQRLRQMLACPPHGLLDRVITNVTIIVLLWAVVWSITGSECLPGGNLFGIII
                                              *:*       *:
                 ↑

                              NSGATSGWYHDFLETPVQLRVGSLEINK
                          TM1
NhaA ————————————RFFSSDASGGIILIIAAILAMIMA                NMLLFWIND——
NapA IFYLLL-AAQVMAFIFK--RLNQPVVIGEVLAGVLVGPALLGLVHEGE-I-LEFLAE---
NHA2 LFYCAIIGGKLLGLIKLPTLPPLPSLLGMLLAGFLIRNIPV--INDNVQIKHKWSSSLRS
     :**  : ..:::.:*           * ::* :***.*:    :  :.:   *  :: :.

        TM2                                          TM3
NhaA ALMAVFFLLVGLEVK———————————————————AFPVIAAIGGMIVPALLYLAFN
NapA LGAVFLLFMVGLETRLKDIL---------AVG------KEAFLVAVLGVALPFLGGYLYG
NHA2 IALSIILVRAGLGLDSKALKKLKGVCVRLSMGPCIVEACTSALLAHYLLGLPWQWGFILG
     :.  ::*. .**   * :         ::*         : *:*  :.**:  *:: *

                  TM4a-b                              TM5
NhaA ————————ITREHWAIPAATDIAFALGVLALLG————ALKIFLMALAIIDDLGAIIIIAL
NapA LEIGFETLPALFLGTALVATSVGITARVLQELGVLSRPYSRIILGAAVIDDVLGLIVLAV
NHA2 FVLGAVS-PAVVVPSMLLLQGGGY--------G-VEKGVPTLLMAAGSFDDILAITGFNT
     : :*  : **:.: : *:  . *         * :.:   :::.*. :**:*.:  : .
```

Figure S8. Comparison of dimer interface composition in NhaA, NapA, and NHA2 proteins. **(A)** In the NhaA crystal structure, the dimer interface is composed of an N-terminal β-hairpin, resulting in minimal promoter contacts between helices (shown in green). In NapA the N-terminal β-hairpin found in NhaA is absent and, instead, extensive interprotomer contacts are formed by an additional N-terminal helix (TM -1) (shown in green) not found in NhaA. **(B)** A structural alignment of NapA and NhaA

shows the position of the additional N-terminal helix in NapA (TM -1). A pairwise sequence alignment between NapA and human NHA2 shows that it also contains residues at the N-terminus that are likely to form an TM segment in the same location as TM-1 in NapA (red boxes). The positions of TM segments in NHA2 (red and green boxes) were predicted from the topology algorithm TMHMM. An arrow indicates the start of the NHA2 construct used in this study.
